# Risk factors associated with morbidity and mortality outcomes of COVID-19 patients on the 28th day of the disease course: a retrospective cohort study in Bangladesh

**DOI:** 10.1017/S0950268820002630

**Published:** 2020-10-29

**Authors:** M. Z. Islam, B. K. Riaz, A. N. M. S. Islam, F. Khanam, J. Akhter, R. Choudhury, N. Farhana, N. A. Jahan, M. J. Uddin, S. S. Efa

**Affiliations:** 1 Department of Community Medicine, National Institute of Preventive and Social Medicine (NIPSOM), Mohakhali, Dhaka 1212, Bangladesh; 2 Department of Public Health and Hospital Administration, National Institute of Preventive and Social Medicine (NIPSOM), Mohakhali, Dhaka 1212, Bangladesh; 3 Department of Parasitology, National Institute of Preventive and Social Medicine (NIPSOM), Mohakhali, Dhaka 1212, Bangladesh; 4 Department of Microbiology and Mycology, National Institute of Preventive and Social Medicine (NIPSOM), Mohakhali, Dhaka 1212, Bangladesh; 5 Department of Nutrition and Biochemistry, National Institute of Preventive and Social Medicine (NIPSOM), Mohakhali, Dhaka 1212, Bangladesh

**Keywords:** Cohort, COVID-19 patients, morbidity, mortality and outcomes, risk factors

## Abstract

Diverse risk factors intercede the outcomes of coronavirus disease 2019 (COVID-19). We conducted this retrospective cohort study with a cohort of 1016 COVID-19 patients diagnosed in May 2020 to identify the risk factors associated with morbidity and mortality outcomes. Data were collected by telephone-interview and reviewing records using a questionnaire and checklist. The study identified morbidity and mortality risk factors on the 28th day of the disease course. The majority of the patients were male (64.1%) and belonged to the age group 25–39 years (39.4%). Urban patients were higher in proportion than rural (69.3% *vs.* 30.7%). Major comorbidities included 35.0% diabetes mellitus (DM), 28.4% hypertension (HTN), 16.6% chronic obstructive pulmonary disease (COPD), and 7.8% coronary heart disease (CHD). The morbidity rate (not-cured) was 6.0%, and the mortality rate (non-survivor) was 2.5%. Morbidity risk factors included elderly (AOR = 2.56, 95% CI = 1.31–4.99), having comorbidity (AOR = 1.43, 95% CI = 0.83–2.47), and smokeless tobacco use (AOR = 2.17, 95% CI = 0.84–5.61). The morbidity risk was higher with COPD (RR = 2.68), chronic kidney disease (CKD) (RR = 3.33) and chronic liver disease (CLD) (RR = 3.99). Mortality risk factors included elderly (AOR = 7.56, 95% CI = 3.19–17.92), having comorbidity (AOR = 5.27, 95% CI = 1.88–14.79) and SLT use (AOR = 1.93, 95% CI = 0.50–7.46). The mortality risk was higher with COPD (RR = 7.30), DM (RR = 2.63), CHD (RR = 4.65), HTN (RR = 3.38), CKD (RR = 9.03), CLD (RR = 10.52) and malignant diseases (RR = 9.73). We must espouse programme interventions considering the morbidity and mortality risk factors to condense the aggressive outcomes of COVID-19.

## Introduction

A newly emergent coronavirus (severe acute respiratory syndrome coronavirus 2 − SARS-CoV-2) causes coronavirus disease 2019 (COVID-19) was first documented in Wuhan City, Hubei Province, China in December 2019 as an outbreak of pneumonia of unknown cause [1]. Based on phylogeny, taxonomy and established practice, on 11 February 2020, the World Health Organization (WHO) named the disease as COVID-19 [2]. WHO declared COVID-19 as a global emergency on 30 January 2020 [3] and as pandemic on 11 March 2020 [4]. Globally 213 countries (on 11 August 2020) are confronting the grave consequences of the ongoing COVID-19 pandemic [5]. The situation is sprouting rapidly with increasing case counts and deaths worldwide [5]. In this course, Bangladesh is also confronting the tolls of morbidity and mortality posed by this highly infectious disease with community transmission across the country.

In our setting, a patient with acute respiratory illness (ARI) and residence of Bangladesh or travel to a country reporting community transmission of COVID-19 disease during the 14 days before symptom onset, or a patient/health care worker with any ARI and having been in contact with a confirmed or probable COVID-19 case in the last 14 days before symptom onset, or a patient with ARI and in the absence of an alternative diagnosis that fully explains the clinical presentation’ is considered as a suspect case. Moreover, a suspect case for whom testing for the COVID-19 virus is inconclusive (inconclusive being the result of the test reported by the laboratory), or a suspected case for whom testing could not be performed for any reason is considered as probable case and a person with laboratory confirmation of COVID-19 infection, irrespective of clinical sign and symptom is known as a confirmed case. According to the guideline, all suspected cases have undergone RT-PCR test for COVID-19 infection [6].

The clinical spectrum of COVID-19 appears to be in a wide range, encompassing asymptomatic infection, mild (40%) or moderate (40%) disease, severe disease (15%) that requires oxygen support and only 5% critical disease [1]. The incubation period of COVID-19 infection is around 5.2 days and the period from the onset of symptoms to death ranges from 6 to 41 days with a median of 14 days [6]. A study conducted in Wuhan of China found that increased age and various comorbidities like hypertension (HTN) and diabetes mellitus (DM) were associated with the severity of COVID-19. But the study did not identify the tobacco use and chronic obstructive pulmonary disease (COPD) as the risk factors for COVID-19 [7].

Another study in China found that nearly half of the patients had comorbidity where HTN was the most common followed by DM and coronary heart disease (CHD). The study also established the association between increased age and death of the COVID-19 patients [8]. Another study showed that severe patients were older and had comorbidities including HTN (30.0%), DM (12.1%) and cardiovascular diseases. The median age was 64 years in severe cases and 51.5 years in non-severe cases [7]. The presence of any comorbidity was more common among the severe patients than those having a mild or moderate disease (38.7% *vs.* 21.0%) with a similar exposure history between the two groups of disease severity [9].

Widespread shreds of evidence have emphasised the harmful impact of tobacco use possibly related to adverse outcomes of COVID-19. One of the leading studies conducted in the Wuhan city of China found higher percentages of current and former tobacco users among the patients that needed ICU support, mechanical ventilation or who had died, and a higher prevalence of smoking among the severe cases [10].

A study conducted on outcomes of the COVID-19 patients found that non-survivors were more often older and men, and they had a higher prevalence of DM, hyperlipidemia and CHDs. The history of current tobacco uses and having COPD was more among the non-survivors [11].

However, the pandemic is still under progression, diverse risk factors influence the outcomes of COVID-19, but relevant data and studies are very scarce in Bangladesh. Therefore, it is irrefutably obligatory to determine the risk factors to avert the aggressive consequences of COVID-19 patients. Based on these realities, in this particular study, we aimed to identify the risk factors associated with morbidity and mortality outcomes of COVID-19 patients.

## Methods

### Study setting, design and population

This observational retrospective cohort study was conducted at the National Institute of Preventive and Social Medicine (NIPSOM), Dhaka, Bangladesh during the period from March to June 2020. The study enrolled a cohort of laboratory-confirmed 1016 COVID-19 (SARS-CoV-2) patients diagnosed at the central laboratory of NIPSOM, Dhaka. The NIPSOM is the apex public health institute holding the central laboratory designated for COVID-19 diagnosis, approved by the Ministry of Health and Family Welfare of the government of Bangladesh. The study included all the hospitalised, non-hospitalised and outdoor patients irrespective of sign/symptom, who were referred to the central laboratory of NIPSOM and diagnosed as COVID-19 by real-time reverse transcriptase-polymerase chain reaction (RT-PCR) assay. The participant who had no contact telephone/cell phone number; who did not respond to a phone call on three separate occasions within 28 days of diagnosis; who were unwilling; and who had incomplete interview were excluded from the study.

### Cohort

The cohort comprised all the laboratory-confirmed COVID-19 patients who were diagnosed at the central laboratory of NIPSOM during the period from 1 to 30 May 2020 by RT-PCR assay of nasopharyngeal (NP)/oropharyngeal (OP)/nasal swab.

### Exposures

The exposures were the risk factors associated with the morbidity and mortality outcomes of COVID-19 patients. It included baseline characteristics, comorbidities like chronic obstructive pulmonary disease, DM, CHDs, HTN, CLDs, CKD, malignant disease, pregnancy and tobacco consumption.

### Outcomes

The study identified both morbidity (cured/not-cured) and mortality (survivor/non-survivor) outcomes of the COVID-19 and compared between exposed and non-exposed groups on the 28th day of the disease course. All the patients underwent the RT-PCR test to evaluate the morbidity status. A patient showing a negative result of the RT-PCR test within 28 days of the disease course was considered as cured. We obtained the RT-PCR test results of the patients from the records preserved by the central laboratory of NIPSOM.

### Sample size and sampling

Initially, we selected all the 1187 laboratory-confirmed COVID-19 patients diagnosed in May 2020 as the study cohort. Finally, 1016 COVID-19 patients were enrolled as the study samples considering the selection criteria and single-centred cluster sampling technique. All the COVID-19 patients formed the sampling frame, and each patient was a sampling unit.

### Data collection and analysis

Data were collected by telephone interview and medical records review using a semi-structured questionnaire and checklist. Each telephone interview session was recorded by a digital recorder to ensure the validity of data. The data collection instruments were pretested on COVID-19 patients diagnosed in April 2020, and accordingly, necessary corrections were performed for finalisation. Participation of COVID-19 patients was voluntary and informed oral consent was obtained from each participant before data collection. In the case of non-survivor, data were collected from the eligible family member of the respective non-survivor. Measures were taken to ensure data quality; inconsistency and irrelevance of data were checked and corrected. Data were analysed using SPSS Statistics (Version 25.0, IBM Statistical Product and Service Solutions, Armonk, NY, USA).

### Statistical analysis

The normality of the variables was tested with the Shapiro−Wilk test/Kolmogorov−Smirnov tests of normality. Continuous data were written in the form of mean and standard deviation. Categorical data were reported as counts and percentages. Descriptive statistics estimated mean, standard deviation and frequency while inferential statistics included chi-square test, logistic regression, relative risk (RR) and attributable risk (AR). A *P*-value <0.05 was considered significant. All the statistical tests were two-sided and were performed at a significance level of *α* = 0.05.

### Measurement of exposure and outcome

Exposures were measured by assessing exposure on the risk factors including baseline characteristics, comorbidity, tobacco consumption, of the COVID-19 patients retrospectively. Outcomes were measured by assessing the morbidity outcome in terms of cured or not-cured and the mortality outcome in terms of survivor or non-survivor.

### Ethics

The study was conducted by maintaining all kinds of ethical issues in different stages of the study. Ethical clearance was obtained from the Institutional Ethics Committee (IEC) of NIPSOM, Dhaka, Bangladesh (Ref. No. NIPSOM/IRB/IEC/2020/1). Informed oral consent was obtained from the participants by informing the purpose and procedure, expected duration, nature and anticipated physical and psychological risks and benefits of participating. The confidentiality of data and privacy of the participants was strictly maintained. The participants were offered the right to withdraw their consent at any stage of the study. Data were stored in computers at the central office, NIPSOM under the direct supervision of the principal investigator. Data were used anonymously for this study only.

## Results

**Fig. 1.**
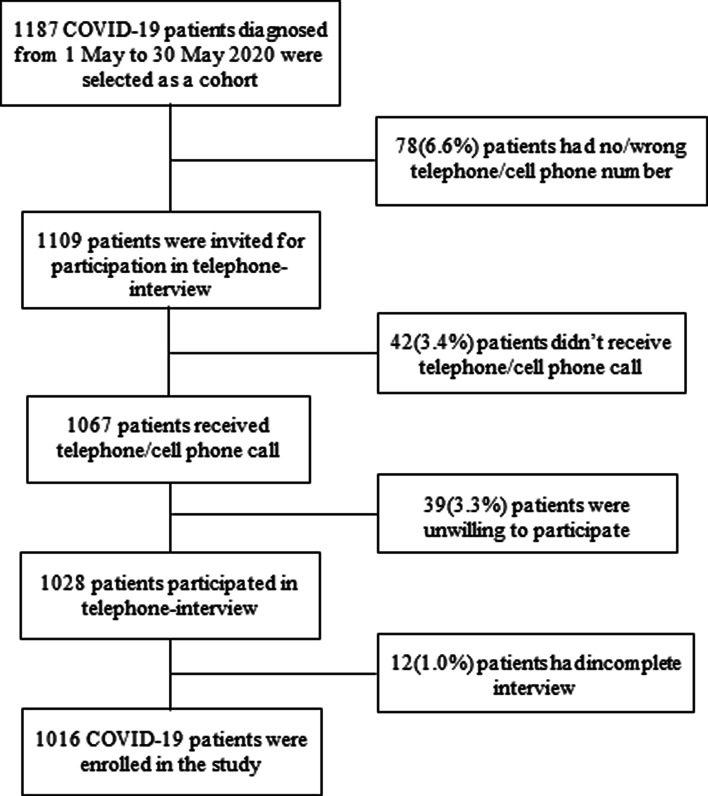
Flow chart of the study participants (COVID-19 patients).

Out of 1187 COVID-19 patients, 1016 (85.6%) were enrolled followed by 6.6% had a wrong contact number, 3.5% did not attend phone calls, 3.3% were unwilling to participate and 1.0% had an incomplete interview (Fig. 1).

### Baseline characteristics of COVID-19 patients

Among 1016 COVID-19 patients, the majority (64.1%) were males (89.9%). The majority (39.4%) of the patients were in the age group 25–39 years, 10.1% belonged to the age group 60–85 years and their median (IQR) age was 37.0 (28–49) years. Of all, 72.6% were married, 39.6% completed their graduation, the majority (32.5%) were service holders and 18.6% were health workers. More than two-thirds (69.3%) was from urban settings, and around three-fourth (72.7%) was from a nuclear family. The majority (37.0%) had monthly family income between TK.10 000 and 30 000, and their average monthly family income was TK.52 859 (±51 000.37) (Table 1).

**Table 1.** Distribution of COVID-19 patients by baseline characteristics (*n* = 1016)

Baseline characteristics	*n* (%)	Statistics
Age (years)		
1–24	165 (16.2)	Median (IQR): 37.0 (28−49)
25–39	400 (39.4)	
40–59	348 (34.3)	
60–85	103 (10.1)	
Sex		
Male	651 (64.1)	
Female	365 (35.9)	
Marital status		
Married	738 (72.6)	
Unmarried	236 (23.2)	
Widow/widower/divorced	42 (4.1)	
Education		
Pre-school and illiterate	78 (7.7)	
Primary	106 (10.4)	
Secondary	238 (23.4)	
Higher secondary	192 (18.9)	
Graduation and above	402 (39.6)	
Occupation		
Housewife	158 (15.6)	
Service	330 (32.5)	
Business	142 (14.0)	
Healthcare worker	189 (18.6)	
Student	104 (10.2)	
Retired/unemployed	76 (7.5)	
Agriculture worker and day labour	17 (1.7)	
Place of residence		
Rural	312 (30.7)	
Urban	704 (69.3)	
Type of family		
Nuclear	739 (72.7)	
Joint	277 (27.3)	
Monthly family income (TK.)		
<10 000	72 (7.1)	Mean (±SD): 52 859 (±51 000.37)
10 000–30 000	376 (37.0)	
31 000–50 000	264 (26.0)	
51 000–100 000	221 (21.8)	
>100 000	83 (8.2)	

TK., Taka, currency of Bangladesh; *n*, number; SD, standard deviation.

### Comorbidity

**Fig. 2.**
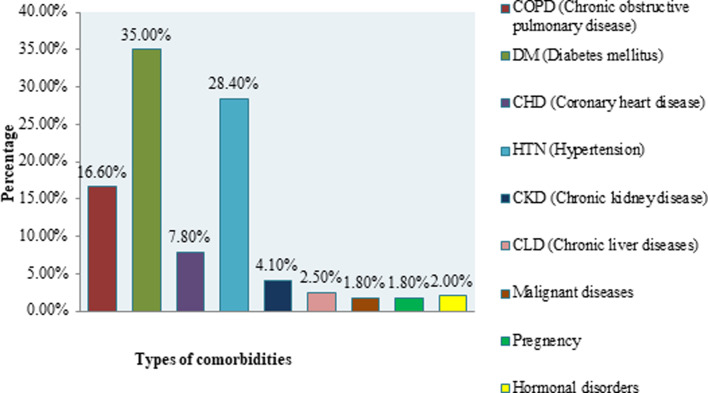
Distribution of COVID-19 patients by types of comorbidities.

More than one third (33.9%) patients had at least one comorbidity. Major comorbidities included DM (35.0%), HTN (28.4%), COPD (16.6%), CHD (7.8%), CLD (2.5%), CKD (4.1%) and malignant diseases (1.8%) (Fig. 2).

### Outcomes of the COVID-19 patients

**Fig. 3.**
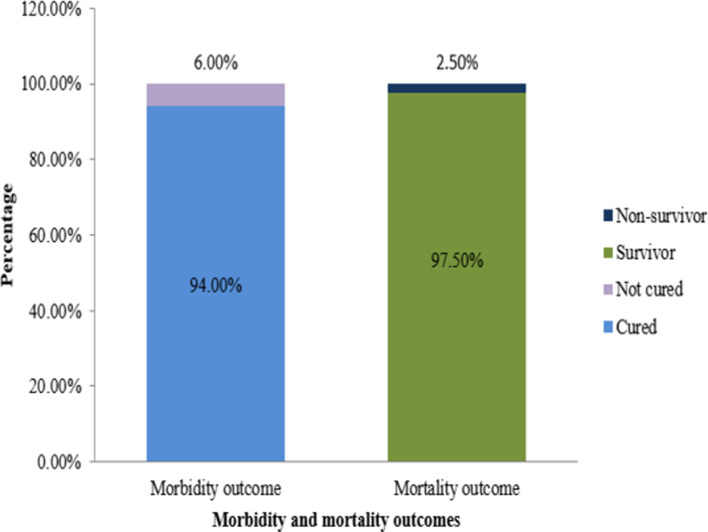
Distribution of COVID-19 patients by morbidity and mortality outcomes.

Morbidity outcomes included 94.0% cured and 6.0% not-cured. On the contrary, mortality outcomes included 97.5% survivors and 2.5% non-survivors (Fig. 3).

### Risk factors associated with outcomes of COVID-19 patients

Regarding risk factors associated with morbidity, the elderly (24.6% *vs.* 9.2%) were significantly (*ρ* < 0.01) higher among the not-cured than the cured patients. Having comorbidity (45.9% *vs.* 33.1%), current SLT use (9.8% *vs.* 3.6%), CKD (6.6% *vs.* 1.8%) and CLD (4.9% *vs.* 1.0%) were also significantly (*ρ* < 0.05) higher among the not-cured than the cured patients. COPD (19.7% *vs.* 7.6%), CKD (6.6% *vs.* 1.8%) and CLD (4.9% *vs.* 1.0%) were also significantly higher among the not-cured than the cured patients (*ρ* < 0.05). Regarding risk factors associated with mortality, the elderly (56.0% *vs.* 9.0%), having comorbidity (80.0% *vs.* 32.7%) and current SLT use (12.0% *vs.* 3.7%) were significantly higher among the non-survivors than the survivors (*ρ* < 0.01). COPD (40.0% *vs.* 7.6%), DM (36.0% *vs.* 17.2%), CHD (16.0% *vs.* 3.6%), HTN (36.0% *vs.* 13.7%), CKD (16.0% *vs.* 1.7%), CLD (12.0% *vs.* 1.0%) and malignant diseases (8.0% *vs.* 0.7%) were also significantly higher among the non-survivors than the survivors (*ρ* < 0.05) (Table 2).

**Table 2.** Risk factors associated with outcomes of COVID-19 patients (On the 28th day)

	Morbidity outcome (*n* = 1016)			Mortality outcome (*n* = 1016)		
Risk factors	Cured *n* (%)	Not cured *n* (%)	*Ρ*-value (95% CI)	Survivors *n* (%)	Non-survivors *n* (%)	*P*-value (95% CI)
Age (years)						
⩽59	867 (90.8)	46 (75.4)	<0.01*	902 (91.0)	11 (44.0)	<0.01**
⩾60	88 (9.2)	15 (24.6)		89 (9.0)	14 (56.0)	
Sex							
Male	609 (63.8)	42 (68.9)	0.422*	632 (63.8)	19 (76.0)	0.208*
Female	346 (36.2)	19 (31.1)		359 (36.2)	06 (24.0)	
Residence						
Rural	290 (30.4)	22 (36.1)	0.350*	304 (30.7)	08 (32.0)	0.887**
Urban	665 (69.6)	39 (63.9)		687 (69.3)	17 (68.0)	
Having symptom during diagnosis	687 (71.9)	45 (73.8)	0.772*	715 (72.1)	17 (68.0)	0.648*
Having comorbidity	316 (33.1)	28 (45.9)	<0.05*	324 (32.7)	20 (80.0)	<0.01*
Current tobacco user	173 (18.1)	12 (19.7)	0.760*	181 (18.3)	4 (16.0)	0.772**
Current SLT user	34 (3.6)	6 (9.8)	<0.05**	37 (3.7)	3 (12.0)	<0.05**
COPD	73 (7.6)	12 (19.7)	<0.01*	75 (7.6)	10 (40.0)	<0.01**
DM	166 (17.4)	13 (21.3)	0.435*	170 (17.2)	09 (36.0)	<0.05**
CHD	35 (3.7)	05 (8.2)	0.086**	36 (3.6)	04 (16.0)	<0.05**
HTN	133 (13.9)	12 (19.7)	0.214*	136 (13.7)	09 (36.0)	<0.01**
CKD	17 (1.8)	04 (6.6)	<0.05**	17 (1.7)	04 (16.0)	<0.01**
CLD	10 (1.0)	03 (4.9)	<0.05**	10 (1.0)	03 (12.0)	<0.01**
Malignant diseases	07 (0.7)	02 (3.3)	0.097**	07 (0.7)	02 (8.0)	<0.01**
Pregnancy	08 (0.8)	01 (1.6)	0.429**	09 (0.9)	00 (0.0)	1.000**
Hormonal diseases	09 (0.9)	01 (1.6)	0.463**	09 (0.9)	01 (4.0)	0.221**

*n*, number; %, percentage; *P* < 0.05, significant; CI, confidence interval; COPD, chronic obstructive pulmonary disease; DM, diabetes mellitus; CHD, coronary heart disease; HTN, hypertension; CKD, chronic kidney disease; CLD, chronic liver disease.

* *χ*^2^-test; **Fisher's exact test.

Logistic regression analysis revealed that the risk of ‘not-cured’ (morbidity) was significantly higher (*P* < 0.05) among the elderly (AOR: 2.56, 95% CI: 1.31–4.99), patients with comorbidity (OR: 1.72, 95% CI: 1.02–2.89) and current SLT users (OR: 2.95, 95% CI: 1.19–7.34). On the contrary, the risk of being ‘non-survivor’ (mortality) outcome was significantly higher among the elderly (AOR: 7.56, 95% CI: 3.19–17.92), patients with comorbidity (AOR: 5.27, 95% CI: 1.88–14.79) and current SLT users (OR: 3.52, 95% CI: 1.01–12.27) (Table 3).

**Table 3.** Logistic regression analysis of the risk factors associated with morbidity (not-cured) and mortality (non-survivor) outcomes of COVID-19 patients

Risk factors	Unadjusted model			Adjusted model		
	OR	95% CI	*P*-value	AOR	95% CI	*P*-value
*Risk factors associated with morbidity outcome (not-cured)*						
Age						
⩽59	Reference			Reference		
⩾60	3.213	(1.72–5.99)	<0.01	2.559	(1.31–4.99)	<0.01
Having comorbidity						
No	Reference			Reference		
Yes	1.716	(1.02–2.89)	<0.05	1.431	(0.83–2.47)	0.198
Current SLT use						
No	Reference			Reference		
Yes	2.955	(1.19–7.34)	<0.05	2.168	(0.84–5.61)	0.110
*Risk factors associated with mortality outcome (non-survivor)*						
Age (Years)						
⩽59	Reference			Reference		
⩾60	12.899	(5.69–29.26)	<0.01	7.555	(3.19–17.92)	<0.01
Having comorbidity						
No	Reference			Reference		
Yes	8.235	(3.06–22.14)	<0.01	5.268	(1.88–14.79)	<0.01
Current SLT use						
No	Reference			Reference		
Yes	3.516	(1.01–12.27)	<0.05	1.924	(0.50–7.46)	0.344

CI, confidence interval; OR, odds ratio; AOR, adjusted odds ratio; SLT, smokeless tobacco.

### Relative risk and attributable risk of outcomes of COVID-19 patients

The risk of ‘not-cured’ outcome was higher among the elderly (RR: 2.89, AR: 9.52%), current SLT users (RR = 2.66, AR: 9.36%), patients with comorbidity (RR: 1.66, AR: 3.23%), COPD (RR: 2.68, AR: 8.85%), CKD (RR: 3.33, AR: 13.32%) and CLD (RR: 3.99, AR: 17.29%). The risk of ‘non-survivors’ outcome was higher among the elderly (RR: 11.28, AR: 12.39%), current SLT users (RR: 3.33, AR: 5.25%), and patients with comorbidity (RR: 7.81, AR: 5.07%), COPD (RR: 7.30, AR: 10.15%), DM (RR: 2.63, AR: 3.12%), CHD (RR: 4.65, AR: 7.85%), HTN (RR: 3.38, AR: 4.37%), CKD (RR: 9.03, AR: 16.94), CLD (RR: 10.52, AR: 20.88%) and malignant diseases (RR: 9.73, AR: 19.94%) (Table 4).

**Table 4.** Risks of morbidity (not-cured) and mortality (non-survivor) outcomes of COVID-19 patients by selected risk factors

Risk factors	RR	AR%
Risk factors linked with morbidity outcome (not-cured)		
Age (⩾60 years)	2.89	9.52
Having comorbidity	1.66	3.23
Current SLT use	2.66	9.36
COPD	2.68	8.85
CKD	3.33	13.32
CLD	3.99	17.29
Risk factors linked with mortality outcome (non-survivor)		
Age (⩾60 years)	11.28	12.39
Having comorbidity	7.81	5.07
Current SLT use	3.33	5.25
COPD	7.30	10.15
DM	2.63	3.12
CHD	4.65	7.85
HTN	3.38	4.37
CKD	9.03	16.94
CLD	10.52	20.88
Malignant diseases	9.73	19.94

RR, relative risk; AR, attributable risk; SLT, smokeless tobacco; COPD, chronic obstructive pulmonary disease; DM, diabetes mellitus; CHD, coronary heart disease; HTN, hypertension; CKD, chronic kidney diseases; CLD, chronic liver diseases.

## Discussion

We conducted this single centred retrospective cohort study to identify the risk factors associated with morbidity and mortality outcomes of COVID-19 patients on the 28th day of the disease course. We followed the case definitions of COVID-19 mentioned in the national guideline of Bangladesh and included the COVID-19 cases confirmed by the RT-PCR test [6]. Major risk factors identified include elderly, comorbidity and tobacco consumption. The study results propose decisive preventive, promotive and curative interventions to combat the worst outcomes of COVID-19.

### Strength and weakness of the study

The current study is the pioneering initiative for the first time carried out in Bangladesh on the risk factors associated with outcomes of COVID-19 patients. This observational retrospective cohort design was scientifically apposite and feasible for identifying multiple risk factors at a single attempt. We included 1016 COVID-19 patients who were confirmed by the NIPSOM laboratory in May 2020, and in this same study period, the total cases were 37 505 in Bangladesh [12]. The sample size was large enough to draw a valid inference on the risk factors linked with the outcomes of COVID-19 patients considering both morbidity and mortality consequences. The morbidity and mortality rates of the disease identified by the study are notable and preserve crucial policy importance. The study results might not reflect the scenario of the whole country as it was a single centred cohort study and included the patients from some specific urban and rural areas. We targeted all the patients confirmed by the central laboratory of NIPSOM in May 2020, irrespective of their sign and severity of symptoms. Therefore, discussion on the various degrees of symptoms of SARS-CoV-2 infection could not be extensive. Despite a few limitations of recall bias that emerged through a telephone-interview and wrong telephone number, the study findings conserve irrefutable policy implications for prevention and control of the morbidity and mortality outcomes of COVID-19.

### Baseline characteristics

Although males (64.1%) have a reasonably higher risk of being affected by COVID-19 than their counterpart females (35.9%), but the study did not find any significant difference in outcomes by gender of the patients. Other studies [8, 9, 13–15] also revealed similar findings where males were being affected more than females. The majority (39.4%) of the patients were in the age group 25–39 years, and 10.1% belonged to the age group of 60–85 years. According to the demographic profile of Bangladesh, the majority (40.07%) of the population belong to the age group of 25–54 years, and 6.42% belong to the age group >65 years [16]. Though the majority of patients belonged to the middle age group, the adverse outcomes were more prevalent in elderly patients (⩾60 years). The prevalence (69.3%) of COVID-19 was found higher in the urban than in rural areas. The unplanned urbanisation, higher population density and industrialisation in the urban communities increase disease transmission. Moreover, more aware urban people undergo laboratory tests for COVID-19 more than the rural people. By occupation, lion shareholders were service holders (32.5%) and health workforce (18.6%). The service holders like bankers, security forces, police and community forces provide various emergency services that are high-risk groups for COVID-19 infection. The health care providers like doctors, nurses and support staff are more vulnerable to COVID-19 as they have to provide healthcare in direct contact with the patients. Besides, less quality and inadequate quantity of personal protective equipment (PPE) also aggravate their vulnerability to the disease.

### Comorbidity and outcomes of COVID-19 patients

Of all, 33.9% patients had diverse comorbidities including DM (35.0%), HTN (28.4%), COPD (16.6%) and CHD (7.8%). Another study conducted in Wuhan, China [8] also identified DM, HTN, COPD and CHD as major comorbidities with COVID-19 patients. In this study, the percentage of not-cured was 6.0%, and the percentage of non-survivors was 2.5% on the 28th day. A study conducted in China revealed different findings, where the death rate was 3.6% among Chinese patients and 1.5% among patients outside China [17]. This discrepancy demands comprehensive comparative studies, but it may be the result of differences in host factors such as body immunity, comorbidity, food habit and agent factors such as genomes, virulence, transmission probability between the geographies.

### Risk factors associated with morbidity outcome

COPD (RR = 2.68, AR% = 8.85) and elderly (AOR = 2.56, RR = 2.89) were significantly associated with the not-cured outcome of morbidity. Other relevant studies [14, 18] found similar findings concerning age, COPD and morbidity outcomes. It is established that poor body immunity of the elderly patients instigates the worse progression and adverse outcomes of COVID-19. Having comorbidity (OR = 1.72, RR = 1.66), current SLT use (OR = 2.96, RR = 2.66), CKD (RR = 3.33) and CLD (RR = 3.99) were found independently associated with the not-cured outcome of morbidity. It could be explained by the fact that comorbidity increases the severity of COVID-19 and prolongs the morbid condition. Although current smoking did not show any significant association with the morbidity/mortality outcome, current SLT use was independently associated with the morbidity outcome of the disease (OR = 2.96, RR = 2.66). It is reported that 27.5% of adult people (26.9% men, 28.1% women) of Bangladesh take *‘zarda’*, *‘khoinee’*, *‘gul’*, *‘sadapata’*, *‘nossi’* and so forth, which are local smokeless tobacco products [19]. Smokeless tobacco induces pathophysiological changes in the upper respiratory tract, which makes the virus more progressive and aggressive to aggravate acute respiratory distress syndrome and morbidity. Further intensive research and analysis could be conducted to establish the scientific arguments on this association in the Indian sub-continent including Bangladesh.

### Risk factors associated with mortality outcome

Mortality outcome was significantly associated with the elderly (AOR = 7.56, RR = 11.28) and having comorbidity (AOR = 5.27, RR = 7.81). Another retrospective study conducted in Wuhan, China [9] also revealed that the higher median age (69.0 years) of the non-survivors than the survivors (52.0 years). Though the majority of the patients of our study were in the middle age group, the mortality rate was higher among the elderly. It is the fact that compromised body immunity of the elderly patients having comorbidity could not win the fight with COVID-19 rather confront the worst outcome. The study also found comorbidity more in the non-survivors (67%) than the survivors (40%). Mortality outcome was also significantly associated with current SLT use (RR = 3.33), COPD (RR = 7.30), DM (RR = 2.63), CHD (RR = 4.65), HTN (RR = 3.38), CKD (RR = 9.03), CLD (RR = 10.52) and malignant diseases (RR = 9.73). These findings were consistent with other relevant studies conducted in different countries [9, 14, 17]. It is evident that the comorbidity deteriorates the defensive mechanism of the patients and worsen the mortality outcome of COVID-19 patients.

### Policy and public health implications

The identified risk factors associated with the outcomes of COVID-19 patients conserve crucial policy implications for the prevention and control of the morbidity and mortality burden of the disease. The study results could contribute to strengthen and reorganise the health care delivery system of the country for providing need-oriented and prioritised services to COVID-19 patients emphasising the disease course and risk factors associated with morbidity and mortality. The study findings could also contribute to devising effective strategies for the provision of comprehensive health care to COVID-19 patients with comorbidity. Policymakers, health care managers and relevant stakeholders may use the study findings to revise the national treatment guidelines considering the risk factors, adverse outcomes and disease course of COVID-19.

## Data Availability

The data for the study is available by contacting the corresponding author upon request.
